# Prevalence and factors associated with underweight, overweight and obesity among 15-49-year-old men and women in Timor-Leste

**DOI:** 10.1371/journal.pone.0262999

**Published:** 2022-02-10

**Authors:** Promit Ananyo Chakraborty, Animesh Talukder, Shams Shabab Haider, Rajat Das Gupta

**Affiliations:** 1 Department of Public Health, North South University (NSU), Dhaka, Bangladesh; 2 School of Population and Public Health, University of British Columbia, Vancouver, British Columbia, Canada; 3 Center for Non-Communicable Diseases and Nutrition, BRAC James P Grant School of Public Health, BRAC University, Mohakhali, Dhaka, Bangladesh; 4 Center for Science of Implementation & Scale Up, BRAC James P Grant School of Public Health, BRAC University, Mohakhali, Dhaka, Bangladesh; 5 Department of Epidemiology and Biostatistics, Arnold School of Public Health, University of South Carolina, Columbia, South Carolina, United States of America; University of Southern Queensland, AUSTRALIA

## Abstract

**Background:**

Underweight and overweight both have a long-lasting significant effect on human health at the individual and population levels. However, in the context of Timor-Leste, a country that achieved independence around two decades ago, there is a severe scarcity of evidence regarding the underweight and obesity burden. We conducted this study to find out the prevalence of underweight, overweight and obesity and their associated factors.

**Methods:**

This study used the nationally representative data of Timor-Leste Demographic Health Survey 2016 data. We conducted descriptive analysis followed by multivariable logistic regression analysis to find out the prevalence and investigate the associated factors. Both crude and adjusted odds ratio of covariates were reported with 95% confidence interval (CI).

**Results:**

This study analyzed the data from a weighted sample of 16,488 Timorese aged 15–49 years. The prevalence of normal weight, underweight, and overweight or obesity were found to be 55.2% (95% CI: 54.2%-56.2%), 25.5% (95% CI: 24.4%-26.7%), and 19.3% (95% CI: 18.3%-20.3%), respectively. For underweight, age, sex, type of settlement (urban/rural), township, and wealth, marital, and educational status were found to have a statistically significant relationship (*p* < 0.05) with Body Mass Index(BMI). After adjustment for the covariates in the logistic regression model age, sex, township, and wealth and marital status were found to be statistically significant correlates (*p* < .05) of underweight. For overweight and obesity, all the background characteristics included in this study (i.e, age, sex, type of settlement, township, and wealth, marital, and educational status) were found to be statistically significant correlates, after adjustment for the covariates.

**Conclusion:**

This study concludes that Timor-Leste has a significant underweight and overweight burden which needs to be addressed through appropriate interventions. Further studies are also warranted to delve deeper into the complex interplay of factors associated with underweight and overweight.

## Introduction

In 2016, over 1.9 billion adults over the age of 18 were overweight or obese. This number has tripled since estimates from 1975 [1]. Overweight and obesity are leading metabolic risk factors for many non-communicable diseases (NCDs), including cardiovascular and chronic respiratory diseases, diabetes, and cancer among others. Altogether NCDs account for 71% of all global deaths, over 85% of premature deaths due to NCDs occur in low- and middle-income countries [2].

Decades ago overweight was considered a problem exclusive to high-income countries. However, in the current global scenario prevalence and distribution of overweight are not confined within high-income countries anymore. According to the 2018 Global Nutrition Report, undernutrition has slightly declined whereas anemia has risen to 32.8% among women [3]. According to the 2017 United Nations International Children’s Emergency Fund (UNICEF)-led State of Food Security and Nutrition report, global undernourishment prevalence has decreased since the early 2000s. However, the decline was less than 20% and has begun to reverse since 2015 [4]. Due to the continuous global increase in overweight prevalence, it now exceeds that of underweight in all regions. The ineffective tackling of the problem of underweight, combined with the encroaching problem of overweight, has left many low- and middle-income nations caught under the weight of the double-burden of malnutrition (DBM) [5]. Countries affected by this double-burden must focus on the health-related consequence of being underweight as well as the increasing prevalence of NCDs associated with being overweight [5]. The co-occurrence of multiple forms of malnutrition across individual life histories, socioeconomic levels, as well as within households and countries remain an area of importance [6, 7].

There are a variety of short-term and long-term factors that lead to malnutrition. The most common driver of undernutrition is poverty [5, 8]. This association results in a feedback loop as poverty is associated with lower education. Especially among women this also leads to chronic malnutrition throughout childhood [8]. A similar feedback loop between childhood and adulthood overweight has also been observed [8]. A major reason causing the weight gain is the imbalance between caloric intake and energy expenditure. Two of the leading causes for this imbalance is the increased availability of energy-dense foods and the physical inactivity associated with sedentary lifestyles [9]. It is interesting to note that the average portion sizes of many packaged and restaurant foods have increased while their costs have decreased. Conversely, the cost of fresh food products has increased [10].

Timor-Leste is an island country in Southeast Asia, which occupies the eastern half of the island of Timor. Timor-Leste’s relatively short history as an independent nation (officially recognized in 2002) includes significant bloodshed and political instability. Recent stability has allowed the nation to refocus on achieving economic solvency and rebuilding and strengthening nationwide infrastructure [11]. Timor-Leste has been classified as a Least Developed Country (LDC) since its inception. However, it is on track to graduate from this status in the coming years [12]. In terms of healthcare, this may be a double-edged sword, as the country heads toward an epidemiological transition in terms of malnutrition. As of 2018, 41.3% of Timorese women of reproductive age were anemic, which makes Timor-Leste a significant outlier at its relative economic level [13]. Compared to global averages this is relatively low. However, this number indicates a five-fold increase among adult women specifically in the last decade [13]. Consequently, despite making some progress in Global Nutrition Targets related to under-five weight, progress towards all other targets including adult obesity and diabetes has been lacking significantly [14].

Despite significant research regarding nutrition, there is a lack of nationally representative data on the prevalence and socio-demographic determinants of underweight, overweight, and obesity in the adult population of Timor-Leste. An updated data on the prevalence and risk factors of underweight, overweight, and obesity will help the policymakers and public health managers design strategies and public health interventions effectively. In 2016, the Timor-Leste Demographic Health Survey (TLDHS) was conducted. It collected nationally representative anthropometric data on the 15–49 years population in Timor-Leste. This study aimed to find out the prevalence and factors associated with underweight, overweight and obesity among 15-49-year-old men and women in Timor-Leste using the TLDHS 2016 data.

## Methods

### Study settings and data source

This study utilized the data of a nationally representative cross-sectional survey called TLDHS 2016. The TLDHS data was collected between September 2016 to December 2016 by the General Directorate of Statistics (GDS) division of the Ministry of Planning and Finance. The financial and technical support of the survey was provided by the ICF and the United States Agency for International Development (USAID). A two-stage stratified cluster random sampling technique was applied for data collection. At the first stage, using total probability proportional to size 455 enumeration areas (EAs) were selected. Among these EAs, 129 were from the urban area and 326 were from the rural area. Then, 26 households were selected randomly from each of the selected EA, yielding a total of 11,829 selected households. Final data were collected from 11,502 households (99% response rate). Among these, 3,215 households were from the urban area and 8,287 households were from the rural area. Men and women aged 15–49 years who were either permanent residents or resided in the households overnight before the survey, were interviewed (97% response rate). The sampling strategy, data collection, and descriptive findings of TLDHS 2016 were published elsewhere [15].

### Data collection, measurements, and quality control

Standard men and women’s questionnaires were used for data collection, which was adapted according to the context of Timor-Leste. The questionnaires were first developed in English, then translated into Tetum language (local language of Timor-Leste). Trained enumerators collected data using tablet computers. Measuring boards and SECA scales (both were calibrated) were used for anthropometric measurements. Survey questionnaire was pre-tested between 7 July to 12 July 2016. Main training took place between 10 August to 13 September 2016. TLDHS technical team as well as DHS program experts conducted the training. Following the training, data collection took place between 16 September to 22 December 2016. Twenty teams were involved in data collection. In each team, there was a supervisor, one editor, one male interviewer, three female interviewer, and one driver. Supervisors and editors were responsible for maintaining data quality at the field level as well as resolving any inconsistencies/confusions. Data were collected electronically through tablet computer, which was transferred daily to central data processing office. The field activity was supervised and coordinated by DHS program staff along with officials from GDS, Ministry of Health, USAID, United Nations Population Fund (UNFPA). Data was processed and checked for inconsistencies, incompleteness, and outliers, and were edited accordingly to ensure completeness of the data. The secondary editing was done at GDS central office which included solving inconsistencies and coding open-ended questions. CSPro software was used for data editing [15].

### Outcome variable

The outcome of interest was the body mass index (BMI) of the participants, which was calculated by dividing the weights in kilogram (kg) by height squared in meter squared (m^2^). An Asia-specific BMI index was used for BMI categorization. The outcome was categorized into following categories: categorize underweight (<18.5 kg/m^2^), normal weight (≥18.5 kg/m^2^ to <23 kg/m^2^), overweight and obesity (≥23 kg/m^2^) [16].

### Explanatory variables

The following covariates were considered based on literature review: (a) age group (15–24 years, 25–34 years, 35–49 years); (b) sex (male, female); (c) place of residence (urban, rural); (d) municipality of residence (Aileu, Ainaro, Baucau, Bobonaro, Covalima, Dili, Ermera, Lautem, Liquiçá, Manatuto, Manufahi, SAR of Oecussi, Viqueque); (e) highest educational status (no formal education, primary, secondary, higher); (f) wealth index (poorest, poorer, middle, richer, richest); (g) marital status (single, currently married, separated/divorced/widowed) [17–19].

The DHS program calculated wealth index based on construction materials for households, water and sanitation facilities, as well as household possession of selected assets including bicycles and television through principle component analysis technique [15]. The wealth index was categorized into quintiles.

## Data analysis

At first, descriptive analyses were conducted, and the findings were reported in frequencies and percentages. Then bivariate analyses were performed between the selected covariates and the BMI status. Finally, bivariate logistics regression analyses were used to identify the factors associated with underweight and overweight/obesity, as compared to normal BMI. The variables which yielded a p-value of <0.2 (which was considered enough to control residual confounding) were included in the final multivariable model [20]. Both crude odds ratio (COR) and adjusted odds ratio (AOR) were reported, along with 95% confidence interval (CI). A p-value <0.05 was considered to be statistically significant. All analyses were done using Stata version 16.0.

## Ethical consideration

TLDHS 2016 study protocol was approved by the ICF Institutional Review Board (ICF IRB FWA00000845). Before data collection, informed consent was collected from the study participants [15]. Permission for using TLDHS 2016 for this study was obtained from DHS program in March 2021. Due to utilization of publicly available and deidentified data in this study, it was deemed exempted from the by the institutions’ Institutional Review Board (IRB).

## Findings

This study analyzed the data from a weighted sample of 16,488 Timorese (aged 15–49 years) to estimate the prevalence of malnutrition and the associated factors among them. The prevalence of normal weight, underweight, and overweight or obesity were found to be 55.2% (95% CI: 54.2%-56.2%), 25.5% (95% CI: 24.4%-26.7%), and 19.3% (95% CI: 18.3%-20.3%), respectively. The description of background characteristics of the respondents as well as the distribution of BMI across different categories of background characteristics are outlined in Table 1.

**Table 1:** Distribution of background characteristics of respondents according to BMI status, (N = 16,488), TLDHS 2016.

Background characteristics	Overall (N = 16,488)		Underweight (N = 4,211)		Normal BMI (N = 9,099)		Overweight / Obesity (N = 3,178)		*p*-value
	n	%	n	%	n	%	n	%	
**Age (in years)**									
15–24	6740	40.9	2424	36.0	3712	55.1	604	9.0	< 0.0001
25–34	4845	29.4	961	19.8	2697	55.7	1187	24.5	
35–49	4903	29.7	826	16.9	2690	54.9	1387	28.3	
**Sex**									
Male	3991	24.2	1015	23.6	2352	50.5	624	26.0	< 0.0001
Female	12497	75.8	3196	26.4	6747	57.3	2554	16.2	
**Settlement**									
Urban	5140	31.2	1211	23.6	2594	50.5	1336	26.0	< 0.0001
Rural	11348	68.8	3000	26.4	6506	57.3	1842	16.2	
**Township**									
Aileu	714	4.3	176	24.6	431	60.4	107	15.0	< 0.0001
Ainaro	711	4.3	156	22.0	441	62.1	113	16.0	
Baucau	1705	10.3	371	21.8	944	55.3	391	22.9	
Bobonaro	1240	7.5	364	29.3	704	56.8	172	13.9	
Cova- Lima	1006	6.1	312	31.0	461	45.8	233	23.2	
Dili	3891	23.6	963	24.8	1933	49.7	994	25.6	
Ermera	1570	9.5	435	27.7	957	61.0	178	11.3	
Lautem	844	5.1	178	21.1	510	60.4	157	18.6	
Liquiçá	1029	6.2	297	28.8	599	58.2	133	13.0	
Manatuto	739	4.5	191	25.9	387	52.4	161	21.7	
Manufahi	936	5.7	218	23.3	550	58.8	168	18.0	
SAR of Oecussi	990	6.0	350	35.4	519	52.4	121	12.3	
Viqueque	1113	6.8	200	18.0	663	59.6	250	22.4	
**Educational status**									
No Formal Education	3165	19.2	736	23.3	1911	60.4	517	16.4	< 0.0001
Primary	2954	17.9	744	25.2	1625	55.0	585	19.8	
Secondary	8606	52.2	2346	27.3	4643	54.0	1617	18.8	
Higher	1763	10.7	385	21.8	921	52.2	458	26.0	
**Wealth index**									
Poorest	2786	16.9	828	29.7	1629	58.5	329	11.8	< 0.0001
Poorer	3166	19.2	862	27.2	1868	59.0	436	13.8	
Middle	3264	19.8	805	24.7	1893	58.0	566	17.3	
Richer	3524	21.4	821	23.3	1912	54.3	790	22.4	
Richest	3748	22.7	895	23.9	1797	47.9	1057	28.2	
**Marital status**									
Single	6380	38.7	2298	36.0	3538	55.5	544	8.5	< 0.0001
Currently Married	9623	58.4	1777	18.5	5296	55.0	2550	26.5	
Separated / Divorced / Widowed	485	2.9	136	28.0	265	54.7	84	17.3	

Abbreviations: N: sample size; n: frequency; BMI: body-mass index; TLDHS: Timor-Leste Demographic and Health Survey

BMI categories: Underweight, BMI < 18.5 Kg/m^2^; Normal BMI, BMI 18.5 - < 23 Kg/m^2^; Overweight or Obesity, BMI ≥ 23 Kg/m^2^.

The majority of respondents were female (75.8%), aged 15–24 years (40.9%), hailed from rural areas (68.8%), and belonged to the Dili (23.6%), the capital city of Timor-Leste. More than half of the respondents were married at the time of the survey, whereas more than one-third were not (38.7%).

The distribution of BMI revealed statistically significant variation (*P* < .05) across different categories of background characteristics. Among the background characteristics, age, sex, type of settlement (urban/rural), township, and wealth, marital, and educational status were found to have statistically significant relationship (*p* < .05) with BMI (Table 1).

In the logistic regression models (Tables 2 & 3), the associated factors of underweight and overweight or obesity were identified after adjusting for the potential confounders. After adjusting for the covariates, apart from the type of settlement (urban/rural) and educational status, the rest of the background characteristics (i.e. age, sex, township, and wealth and marital status), were found to be statistically significant correlates (*p* < .05) of underweight (Table 2). The older age groups were found to have lower adjusted odds of being underweight (aged 25–34 years: AOR: 0.7, 95% CI: 0.6–0.7; aged 35–49 years: AOR: 0.6, 95% CI: 0.5–0.7, *p* < .001) compared to the 15–24-year-old age-group. Females were found to have 20% higher odds of being underweight than males (AOR: 1.2, 95% CI: 1.1–1.3, *p* < .001). In terms of wealth status, compared to the ‘poorest’ quintile, those who belonged to the higher quintiles had lower odds of being underweight. Also, a statistically significant association was found for all the quintiles except in the case of the ‘poorer’ group. The Timorese who were married at the time of the survey, were found to have 30% lower odds of being underweight (AOR: 0.7, 95% CI: 0.6–0.8, *p* < .001) than the ones who were not married. The same pattern echoed for the ones who were separated, divorced, or widowed, although a statistically significant association was not found in that case (Table 2).

**Table 2:** Crude and adjusted odds ratio (95% CI) estimates of underweight compared to normal weight by respondent background characteristics, TLDHS 2016.

Covariates	COR (95% CI)	*p*-value	AOR (95% CI)	*p*-value
**Age (in years)**				
15–24	Reference		Reference	
25–34	0.5 (0.5–0.6)	< 0.0001	0.7 (0.6–0.7)	< 0.0001
35–49	0.5 (0.4–0.5)	< 0.0001	0.6 (0.5–0.7)	< 0.0001
**Sex**				
Male	Reference		Reference	
Female	1.2 (1.1–1.3)	< 0.0001	1.2 (1.1–1.3)	< 0.0001
**Settlement**				
Urban	1.0 (0.9–1.1)	0.813	Not included in the final model	
Rural	Reference			
**Township**				
Aileu	Reference		Reference	
Ainaro	0.9 (0.7–1.2)	0.364	0.9 (0.7–1.2)	0.566
Baucau	1.0 (0.7–1.2)	0.736	1.0 (0.7–1.2)	0.749
Bobonaro	1.3 (1.0–1.6)	0.062	1.3 (1.0–1.7)	0.022
Cova-Lima	1.7 (1.4–2.2)	< 0.0001	1.9 (1.4–2.4)	< 0.0001
Dili	1.3 (1.0–1.6)	0.021	1.3 (1.0–1.7)	0.027
Ermera	1.1 (0.9–1.4)	0.453	1.1 (0.9–1.5)	0.36
Lautem	0.9 (0.7–1.2)	0.43	0.9 (0.7–1.2)	0.61
Liquiçá	1.3 (1.0–1.7)	0.022	1.4 (1.1–1.8)	0.011
Manatuto	1.2 (0.9–1.6)	0.127	1.3 (1.0–1.7)	0.044
Manufahi	1.0 (0.8–1.3)	0.72	1.1 (0.8–1.4)	0.64
SAR of Oecussi	1.7 (1.3–2.2)	< 0.0001	1.8 (1.4–2.4)	< 0.0001
Viqueque	0.7 (0.6–1.0)	0.025	0.8 (0.6–1.0)	0.048
**Educational status**				
No Formal Education	Reference		Reference	
Primary	1.2 (1.0–1.4)	0.007	1.1 (0.9–1.2)	0.277
Secondary	1.3 (1.2–1.5)	< 0.0001	1.0 (0.9–1.1)	0.866
Higher	1.0 (0.9–1.2)	0.62	0.9 (0.8–1.1)	0.347
**Wealth index**				
Poorest	Reference		Reference	
Poorer	0.9 (0.8–1.0)	0.147	0.9 (0.8–1.0)	0.115
Middle	0.9 (0.7–1.0)	0.013	0.8 (0.7–0.9)	0.003
Richer	0.9 (0.8–1.0)	0.035	0.8 (0.7–0.9)	0.002
Richest	1.0 (0.8–1.1)	0.549	0.9 (0.7–1.0)	0.045
**Marital status**				
Single	Reference		Reference	
Currently Married	0.5 (0.5–0.6)	< 0.0001	0.7 (0.6–0.8)	< 0.0001
Separated / Divorced / Widowed	0.7 (0.6–0.9)	0.004	0.9 (0.7–1.2)	0.608

AOR: Adjusted Odds Ratio; CI: Confidence Interval; COR: Crude Odds Ratio; TLDHS: Timor-Leste Demographic and Health Survey

**Table 3:** Crude and adjusted odds ratio (95% CI) estimates of overweight/obesity compared to normal weight by respondent background characteristics, TLDHS 2016.

Covariates	COR (95% CI)	*p*-value	AOR (95% CI)	*p*-value
**Age (in years)**				
15–24	Reference		Reference	
25–34	2.9 (2.6–3.2)	< 0.0001	1.9 (1.7–2.2)	< 0.0001
35–49	3.6 (3.2–4.0)	< 0.0001	2.3 (2.0–2.7)	< 0.0001
**Sex**				
Male	Reference		Reference	
Female	1.4 (1.3–1.6)	< 0.0001	1.4 (1.3–1.6)	< 0.0001
**Settlement**				
Urban	0.6 (0.5–0.7)	< 0.0001	0.8 (0.7–0.9)	< 0.0001
Rural	Reference		Reference	
**Township**				
Aileu	Reference		Reference	
Ainaro	1.1 (0.8–1.5)	0.466	1.0 (0.8–1.3)	0.853
Baucau	1.7 (1.3–2.2)	< 0.0001	1.3 (1.0–1.7)	0.024
Bobonaro	1.0 (0.8–1.4)	0.796	0.8 (0.6–1.1)	0.118
Cova-Lima	2.0 (1.5–2.6)	< 0.0001	1.5 (1.1–1.9)	0.003
Dili	2.0 (1.6–2.6)	< 0.0001	1.1 (0.9–1.4)	0.348
Ermera	0.8 (0.6–1.1)	0.2	0.8 (0.6–1.0)	0.067
Lautem	1.3 (1.0–1.8)	0.054	1.0 (0.8–1.3)	0.921
Liquiçá	1.0 (0.7–1.3)	0.854	0.8 (0.6–1.1)	0.162
Manatuto	1.7 (1.3–2.3)	< 0.0001	1.3 (1.0–1.7)	0.054
Manufahi	1.3 (1.0–1.7)	0.069	1.1 (0.8–1.4)	0.508
SAR of Oecussi	1.0 (0.7–1.4)	0.91	0.8 (0.6–1.0)	0.063
Viqueque	1.5 (1.1–2.0)	0.005	1.3 (1.0–1.7)	0.044
**Educational status**				
No Formal Education	Reference		Reference	
Primary	1.3 (1.2–1.5)	< 0.0001	1.4 (1.2–1.6)	< 0.0001
Secondary	1.1 (1.0–1.3)	0.06	1.3 (1.2–1.5)	< 0.0001
Higher	1.5 (1.3–1.8)	< 0.0001	1.3 (1.1–1.6)	0.006
**Wealth index**				
Poorest	Reference		Reference	
Poorer	1.2 (1.0–1.4)	0.077	1.2 (1.0–1.4)	0.057
Middle	1.4 (1.2–1.7)	< 0.0001	1.4 (1.2–1.6)	< 0.0001
Richer	2.0 (1.7–2.4)	< 0.0001	1.8 (1.5–2.1)	< 0.0001
Richest	2.8 (2.4–3.2)	< 0.0001	2.3 (1.9–2.8)	< 0.0001
**Marital status**				
Single	Reference		Reference	
Currently Married	3.5 (3.1–3.9)	< 0.0001	2.2 (1.9–2.5)	< 0.0001
Separated / Divorced / Widowed	2.2 (1.7–2.9)	< 0.0001	1.3 (1.0–1.7)	0.068

AOR: Adjusted Odds Ratio; CI: Confidence Interval; COR: Crude Odds Ratio; TLDHS: Timor-Leste Demographic and Health Survey

On the other hand, in the matter of overweight and obesity, all the background characteristics included in this study were found to be statistically significant correlates, after adjustment for the covariates (Table 3). In comparison with the 15–24-year-old age group, the older age groups were found to have around two times higher odds of being overweight or obese (aged 25–34-years: AOR: 1.9, 95% CI: 1.7–2.2, *p* < .001; aged 35–49 years: AOR: 2.3, 95% CI: 2.0–2.7, *p* < .001). Females were found to have 40% higher odds of being overweight or obese than males (AOR: 1.4, 95% CI: 1.3–1.6, *P* < .001). The urban Timorese had 20% lower odds of being overweight or obese than the rural compatriots (AOR: 0.8, 95% CI: 0.7–0.9, *p* < .001. In terms of educational status, compared to the Timorese having no formal education, those with higher educational attainment had significantly higher odds of being overweight or obese. As to wealth status, compared to the ‘poorest’ quintile, those who belonged to the higher quintiles had higher odds of being overweight or obese; the odds in the ‘richest’ quintile were more than twice as high as that of the ‘poorest’ (AOR: 2.3, 95% CI: 1.9–2.8, *p* < .001). The Timorese who were married at the time of the survey were found to have greater than twice the odds of being overweight or obese (AOR: 2.2, 95% CI: 1.9–2.5, *p* < .001) compared to the ones who were not married. The same pattern echoed for the ones who separated, divorced, or widowed, although a statistically significant association was not found (Table 3).

## Discussion

To the best of our knowledge, this study is the first one to provide evidence regarding the nationwide prevalence and correlates of underweight, overweight and obesity among 15-49-year-old men and women in Timor-Leste. To ensure optimum generalizability of our findings, we have used nationally representative data from the Timor-Leste Demographic Health Survey (TLDHS) 2016. Specifically, this study has precisely delineated the prevalence of normal weight, underweight, and overweight or obesity to be 55.2% (95% CI: 54.2%-56.2%), 25.5% (95% CI: 24.4%-26.7%), and 19.3% (95% CI: 18.3%-20.3%) among the respondents. Likewise, it revealed that age, sex, township, and wealth and marital status are significant covariates of underweight. Furthermore, it reported age, sex, township, type of settlement, education, wealth and marital status to be significant covariates of overweight or obesity. We expect this study to provide the policymakers with timely evidence regarding the burden of overweight and obesity in the age group of 15–49 years.

The prevalence of underweight, 25.5% (95% CI: 24.4%-26.7%), was alarmingly high compared to Indonesia (11.2%)–which is also an island country that occupied Timor-Leste for more than two decades [21]. However, compared to another Asian country Bangladesh, the prevalence was lower(30.4%). On the other hand, the prevalence of overweight or obesity,19.3% (95% CI: 18.3%-20.3%), was lower compared to that of Indonesia (30.4%), Malaysia (50.2%), Thailand (40.9%), and Bangladesh (23.5%) [19, 21–23]. That said, one important aspect that might explain the discrepancies in prevalence is that in our study the age range of the study participants was from 15–59 years, whereas the studies conducted in other countries considered a different age range. Although all of the aforementioned countries are in Asia, they differ in many socio-geographical aspects that includes differences in health policies and behaviors. This, too, might explain the difference in prevalence rates of underweight, overweight or obesity.

Our study revealed that 21.8% of the respondents received no formal education. As the majority (68.8%) of the respondents hailed from rural (68.8%) areas, it is not possible to ascertain if the proportion of people with no formal education is comparatively lower among the urban population. However, it is probable that there has been an under-ascertainment, as the true proportion of people with no formal education could be slightly higher. Because the majority of the respondents in this survey was people aged 15–24 years (40.9%) and this age group is likely to have higher proportion of formally educated people compared to their older counterparts. Consequently, it could slightly inflate the overall prevalence of people with no formal education.

In our study, Females were found to have 20% higher odds of being underweight than males. This finding is congruent with two similar studies carried out using nationally representative data in the context of Indonesia and Bangladesh [19, 21]. We have also found that those who belonged to the higher wealth quintiles had lower odds of being underweight. This finding is supported by a previous study carried out in the context of Bangladesh where they reported that children from households in the highest wealth index quintile had lower odds of being underweight (OR  =  0.44, 95% CI: 0.37, 0.53) compared to children from households in the lowest quintile [24]. Furthermore, another study from Indonesia buttressed our findings [21].

Interestingly, our study revealed that Timorese who were married at the time of the survey were found to have 30% lower odds of being underweight. A previous study in the context of Bangladesh echoed our findings as they reported, through a pooled analysis, that not being married was positively associated with being underweight [25]. Two other previous studies in the context of Ethiopia and Iran also supported our findings [26, 27]. In many developing countries, being married provides women with greater financial stability which in turn could work as a protective factor from being underweight. Some other factors such as usage of contraceptive pills, weight gain in the postpartum phase etc. are more likely to be prevalent among married women in many countries’ contexts [28, 29]. Although these are plausible hypotheses that might explain the phenomena, a proper factual explanation warrants a deeper exploration of the socio-cultural dynamics of Timor Leste. Further study to explore this would also either help establish or refute any plausible causal connection between being married and having lower odds of underweight.

A previous study from Bangladesh mimicked our finding regarding females having 40% higher odds of being overweight or obese [19]. Our study also noted that urban Timorese had 20% lower odds of being overweight or obese than rural compatriots. However, a study by *Hashan* et al reported in the context of Malaysia that urban or rural residence was not correlated with being overweight/obese. That said, they found a slightly higher prevalence of overweight/obesity among rural women compared to urban women [17]. This contradiction of findings can be explained by the fact that the demarcation between rural and urban places is not distinct or constant across countries. For example, in some countries, rural and urban women have almost equal access to outdoor physical activities, nutritious foods, and healthcare facilities. However, in some developing countries, rural women are socially expected to work hard in order to complete household chores [30]. They also face more barriers to outdoor physical activities or local gymnasium compared to their urban counterparts [31]. Therefore, the heterogeneity in the difference between rural and urban places, the social expectations from women, and access to physical activities might be some of the issues that explain the contradiction in findings. Our study also found higher odds of obesity/overweight among those who belonged to higher wealth quantile and had higher educational attainment. *Templin* et al reported, through using 182 Demographic and Health Surveys and World Health Surveys, that low-income countries have a higher prevalence of overweight/obesity among wealthier individuals [32]. *Cohen* et al systematically analyzed 289 articles from 91 countries and concluded that a positive correlation between higher educational attainment and obesity was more common in lower-income countries [33]. Lastly, we reported that Timorese who were married had greater than twice the odds of being overweight or obese. This evidence is in line with a previous study from Poland which reported similar findings [34].

Government and stakeholders need to take multilayer obesity preventive initiatives including legislative approaches. Additionally, multisectoral initiatives containing pragmatic yet culturally appropriate strategies need to be formulated in order to achieve the objective. Similarly, policy initiatives and effective interventions in healthcare settings are important in order to effectively tackle the issue of malnutrition.

## Strengths and limitations

This remains, to date, the first study that leveraged a nationally representative dataset to explore the correlates of underweight, and overweight or obesity in the context of Timor-Leste. We have used an Asia specific BMI index to categorize the BMI which helped us increase the precision of our findings. Given the lack of existing scientific evidence in Timor-Leste, this study will build the base of further research which would in-depth explore these associated factors.

The dataset we used did not have several important socio-behavioral and clinical covariates which are known to be associated with underweight and overweight or obesity. As such, we were not able to explore their association with our outcome variables of interest. Furthermore, the absence of including these variables in our logistic regression model could fail to block the confounding pathways and backdoor paths which in turn could confound our effect estimate. Finally, although the male-female ratio in Timor-Leste is 1.03:1.00, the male-female ratio in our study was 1:3 [35]. This is probably due to the primary target population of TLDHS was the women of the reproductive age group [15]. As a result, the estimated prevalence may not represent the nationally-representative prevalence.

## Conclusion

Findings from this study depict a comprehensive picture of the status quo of the burden, distribution, and determinants of underweight, overweight or obesity. We suggest further studies should be carried out to: explore the germane covariates, casually investigate the explanatory-outcome variable relationships, and inspect the socio-cultural and clinical context-specific issues that might affect the prevalence. We further urge that further in-depth studies be carried out using novel approaches building on the evidence reported by our study.
